# An ultra-sensitive and high-throughput trapping-micro-LC-MS method for quantification of circulating vitamin D metabolites and application in multiple sclerosis patients

**DOI:** 10.1038/s41598-024-55939-0

**Published:** 2024-03-06

**Authors:** Flora Qu, Ming Zhang, Bianca Weinstock-Guttman, Robert Zivadinov, Jun Qu, Xiaoyu Zhu, Murali Ramanathan

**Affiliations:** 1 Department of Biochemistry, University at Buffalo, State University of New York, Buffalo, NY USA; 2 Department of Pharmaceutical Sciences, University at Buffalo, State University of New York, Buffalo, NY USA; 3 Department of Neurology, Jacobs School of Medicine and Biomedical Sciences, Jacobs Comprehensive MS Treatment and Research Center, University at Buffalo, State University of New York, Buffalo, NY USA; 4 Buffalo Neuroimaging Analysis Center, Department of Neurology, Jacobs School of Medicine and Biomedical Sciences, University at Buffalo, State University of New York, Buffalo, NY USA; 5 Center for Biomedical Imaging at the Clinical Translational Science Institute, University at Buffalo, State University of New York, Buffalo, NY USA; 6 New York State Center of Excellence in Bioinformatics & Life Sciences, Buffalo, NY USA

**Keywords:** Mass spectrometry, Analytical biochemistry, Liquid chromatography

## Abstract

Quantitative analysis of the biologically-active metabolites of vitamin D (VitD), which are crucial in regulating various physiological and pathological processes, is important for clinical investigations. Liquid chromatography-tandem mass spectrometry (LC-MS) has been widely used for this purpose but existing LC-MS methods face challenges in achieving highly sensitive and accurate quantification of low-abundance VitD metabolites while maintaining high throughput and robustness. Here we developed a novel pipeline that combines a trapping-micro-LC-(T-µLC) with narrow-window-isolation selected-reaction monitoring MS(NWI-SRM) for ultra-sensitive, robust and high-throughput quantification of VitD metabolites in serum samples after derivatization. The selective-trapping and delivery approach efficiently removes matrix components, enabling high-capacity sample loading and enhancing sensitivity, throughput, and robustness. The NWI-SRM further improves the sensitivity by providing high selectivity. The lower limits of quantification (LOQs) achieved were markedly lower than any existing LC-MS methods: 1.0 pg/mL for 1,25(OH)_2_D3, 5.0 pg/mL for 24,25(OH)_2_D3, 30 pg/mL for both 25(OH)D2 and 25(OH)D3, all within a 9-min cycle. The method is applied to quantify VitD metabolites from 218 patients with multiple sclerosis. This study revealed negative correlations(r=− 0.44 to − 0.51) between the levels of 25(OH)D2 and all the three D3 metabolites in multiple sclerosis patients.

## Introduction

Vitamin D (VitD) plays a crucial role in a wide variety of biological processes, such as calcium and bone homeostasis, immunomodulation, and cell differentiation^1^. Circulating 25-hydroxy VitD (25(OH)D), the major circulating metabolite of VitD, is a key marker for VitD deficiency^2^. The most important biologically-active metabolite, 1,25-dihydroxyvitamin VitD (1,25(OH)_2_D), is derived from 25(OH)D^3^. Another di-hydroxyl metabolite, 24,25-dihydroxyvitamin VitD (24,25(OH)_2_D), is the most abundant metabolite of 25(OH)D, and is important in studying diseases related to VitD catabolism disorder^4,5^. VitD has two main forms, D2 and D3. In humans, while D3 can be obtained through cutaneous synthesis or exogenous sources, D2 is only obtained from specific exogenous sources such as fungus^6^. In clinical practice, VitD supplementation is used to treat VitD deficiency^7–9^. While D3 is the main type of VitD supplement on the market, D2 is used substantially, for example, by vegans, due to its non-animal source, and in situations where prescribed high-dosage VitD is necessary to treat certain conditions (*e.g.,* severe osteoporosis, hypoparathyroidism, familial hypocholesterolemia and refractory rickets) because the only FDA-approved high-dosage VitD is in the D2 form^10,11^. The equivalency of D2 and D3, and their metabolites, in terms of biological effects, remains controversial^6,12,13^. For example, a recent blood transcriptomic study reveals that D2 and D3 may have considerably diverse effects on the human immune system ^14^, while different pharmacokinetic properties and biological effects of the metabolites of D2 vs. D3 have been observed^15,16^. Distinct gene transcription, protein expression and cellular responses in human preosteoblasts upon treatment of 1,25(OH)_2_D2 and 1,25(OH)_2_D3 were found^17,18^. Therefore, it is crucial to accurately quantify the metabolites of D2 and D3 in order to comprehensively understand the effects of VitD.

Nonetheless, although 25(OH)D3 can be readily quantified due to its relatively high abundance in circulation, other VitD metabolites, such as the di-hydroxyl-metabolites, are challenging to be quantified with sufficient sensitivity and high accuracy because of their low circulating concentrations^19^. Currently, immunoassays account for more than 90% of the clinical analyses of VitD metabolites, primarily owing to their high throughput^20^. However, immunoassay methods may be susceptible to problems associated with suboptimal specificity, accuracy and precision^21,22^. As a promising alternative, LC-MS-based methods can reliably measure D2 and D3 metabolites because of their high selectivity with a molecular-level resolution. Nevertheless, achieving highly sensitive and accurate quantification of VitD metabolites while maintaining high throughput and robustness remains challenging for LC-MS-based methods^21,23,24^. Although derivatization is largely employed to enhance the ionization efficiency of VitD metabolites, enabling more sensitive LC-MS analysis^25^, the improvement in sensitivity may not be sufficient for measuring low-abundance VitD metabolites, especially in individuals with specific diseases where serum levels of these metabolites are anticipated to be lower than in healthy subjects^26^. To boost the sensitivity, our group has previously described a strategy using a one-step derivatization with a Cookson-type reagent followed by selective solid-phase extraction and micro-flow LC-MS (µLC-MS) for sensitive quantification of VitD metabolites^27,28^. The drawback of the method is relatively long analytical cycles and suboptimal robustness, necessitating the cleaning of the LC-MS system after approximately every 200 runs, which limits its application in large clinical cohorts.

To achieve high-throughput and robust analysis of key VitD metabolites with ultra-high sensitivity, we developed a new pipeline in this study, which combines a trapping-micro-LC (T-µLC) method with narrow-window-isolation selected-reaction monitoring MS (NWI-SRM) for analysis of VitD metabolites in serum samples after derivatization. Previously, we developed a T-µLC-MS method for targeted quantification of proteins^29^. In brief, the system consists of a high-flow LC for rapid online trapping of digested peptide samples on a large-capacity trapping column, and a synchronized low-flow µLC-MS for sensitive analysis^29^. Here we modified and further developed the T-µLC method for ultra-sensitive, robust and high throughput quantification of VitD metabolites in serum samples after derivatization. Moreover, for SRM MS detection, we found that utilizing a narrower *m/z* window at 0.2 Th for precursor isolation on Q1, *i.e.* the NWI-SRM for MS detection, *was synergistic with the T-µLC approach*, largely decreasing co-eluted interferences from samples and thereby enhancing both the sensitivity and selectivity for VitD metabolites.

The optimized T-µLC-NWI-SRM system showed salient advantages over the conventional LC-MS-based methods: (i) by combining a selective trapping/delivery approach on the LC part and the NWI-SRM on the MS part, the method substantially improved signal-to-noise (S/N) ratio; (ii) the large-I.D. trap allowed a high-capacity loading, enhancing the signals of VitD metabolites; (iii) the trapping/delivery approach prevented hydrophobic/hydrophilic matrix components from entering the LC-MS, affording exceptional robustness; and *iv*) the rapid sample trapping and the much simplified matrix for µLC separation facilitated high-throughput analysis. Collectively, this strategy allowed ultra-sensitive, robust, and high-throughput quantification of multiple D2/D3 metabolites, with the LOQs down to 1.0 pg/mL for 1,25(OH)_2_D3, 5.0 pg/mL for 24,25(OH)_2_D3, 30 pg/mL for 25(OH)D2 and 25(OH)D3 within a 9-min analytical cycle.

We applied the established strategy to the quantification of four VitD metabolites in serum samples from **218** multiple sclerosis patients, including 25(OH)D2, 25(OH)D3, 1,25(OH)_2_D3 and 24,25(OH)_2_D3. It was reported that the onset and progression of multiple sclerosis, a chronic central nervous system disease, were associated with the level of serum VitD metabolites^30^. Notably, low blood 25(OH)D levels have been widely reported in multiple sclerosis patients under active disease states^31,32^. Elevated serum 1,25(OH)_2_D levels are correlated with a decreasing risk of developing multiple sclerosis^33^, whereas lower concentrations of the 24,25(OH)_2_D are associated with increased disability in multiple sclerosis patients^28^. Therefore, VitD supplementation is often recommended for multiple sclerosis patients^34^. Accurate measurement of the levels of VitD metabolites in multiple sclerosis patients will facilitate the interpretation of clinical data and guide disease treatment and management. Moreover, examining the correlation of D2 and D3 metabolites in multiple sclerosis patients would provide valuable insights into VitD metabolism in the patient population, but this remains unexplored. With the developed T-µLC-NWI-SRM method, we successfully quantified all metabolites in all samples, with high throughput exceptional sensitivity, accuracy and reproducibility. Intriguingly, we observed a negative correlation between the levels of 25(OH)D2 and the levels of 25(OH)D3, 1,25(OH)_2_D3 and 24,25(OH)_2_D3 in multiple sclerosis patients, an interesting observation meriting further investigation.

## Results

### Development of trapping-micro-LC (T-μLC) strategy for ultra-sensitive, robust and high-throughput quantification of VitD metabolites

**Figure 1:**
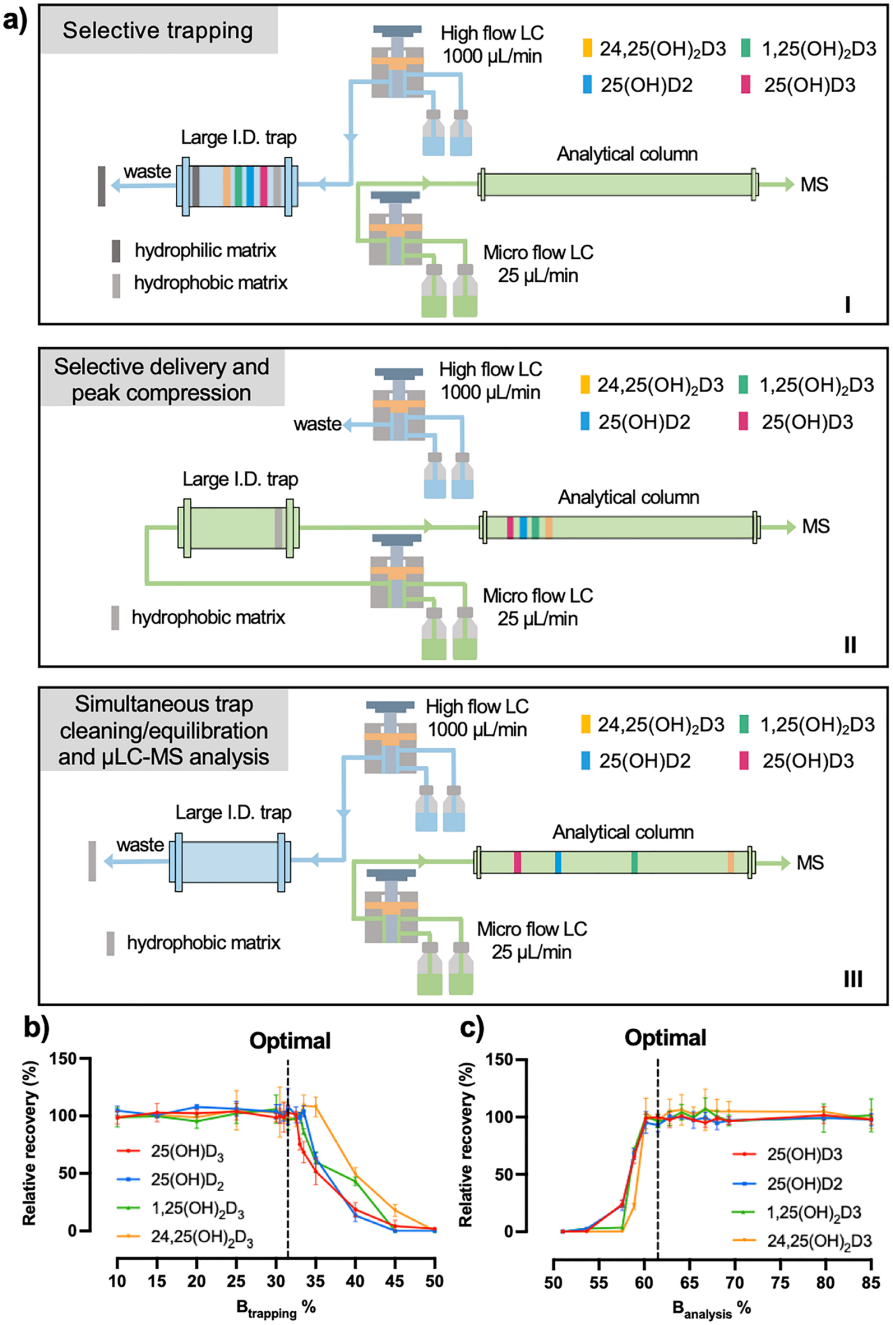
The design and optimization of a trapping-micro-LC-MS (T-µLC-MS) strategy for ultra-sensitive, high-throughput and robust quantification of VitD metabolites. (**a**) the steps involved in the analysis of the VitD metabolites by T-µLC-MS. (**b**) the optimization of B_trapping_% for selective trapping of the VitD metabolites and (**c**) the optimization of the B_analysis_% for selective delivery of the VitD metabolites from the trap to the µLC column, using a pooled human serum sample spiked with VitD metabolites.

As shown in Fig. 1a, the T-μLC system consists of two synchronized LC components: a high-flow LC for fast and large-capacity sample loading and a low-flow LC for sensitive analysis, enabling highly sensitive, robust and high-throughput quantification of VitD metabolites via three steps:

(I) *Selective, rapid trapping* at a high-flow rate of 1000 μL/min on the large I.D. trap, while the trap is not engaged with the analytical column. With an optimized composition of loading mobile phase, the trap selectively concentrates the VitD metabolites from samples, while simultaneously removing more hydrophilic matrix components (*e.g.* salts and polar organic compounds).
(II) *Selective delivery and peak compression.* The trap is switched in line with the analytical column (i.e., the μLC column) and then a microflow gradient at 25 μL/min is used to back-flush the trap, which selectively delivers the VitD metabolites into the μLC-MS system. To prevent peak broadening during this process, two strategies were taken: firstly, we used weaker retention of the trap (C8 stationary phase) vs. analytical column (C18 stationary phase) to enable peak compression when target metabolite is delivered from the trap to column; secondly, retrograde trapping and delivery flows prevent targets from traveling the relatively large trap volume and enable rapid delivery of target metabolites to the μLC column. These two approaches enable effective focusing the VitD metabolites at the front of the analytical column. As soon as the four VitD metabolites have been completely eluted to the front of the analytical column, the trap, carrying matrix components that are more hydrophobic than the four VitD metabolites, is switched off the analytical column. In combination with selective trapping, this approach prevents hydrophilic and hydrophobic matrix components from entering the μLC-MS system, Therefore, the μLC-MS only analyzes the fraction containing concentrated VitD metabolites, enabling ultra-sensitive, robust and rapid quantification.
(III) *Simultaneous trap cleaning/equilibration and μLC-MS analysis.* The trap has been taken offline and is flushed with a loading mobile phase containing a high composition of organic solvent at a rate of 1000 μL/min to remove the trapped hydrophobic matrix components followed by equilibration of the trap. Simultaneously, the μLC-MS is performing a sensitive analysis of the VitD metabolites.

For μLC-MS, we selected a 0.5 mm i.d. C18 column for a balance of sensitivity, robustness, and throughput, and a 3.5 μm material which provided optimal robustness, reproducibility, and separation. A flow rate of 25 μL/min was determined as optimal for μLC, considering resolution, sensitivity, speed, back pressure, and system dead time. For trapping, a 2.1 mm i.d. C8 column was chosen to enhance quantitative loading capacity, with an optimal flow rate of 1 mL/min for fast sample loading, matrix component removal, and equilibration. The key parameters of the procedure were meticulously optimized to maximize sensitivity, reproducibility and robustness. The T-μLC-MS gradients, as wells as procedures for PTAD derivatization of VitD metabolites are described in “Method”. The MS transitions of each target are shown in Supplementary Table 1.

#### Substantially increased sensitivity by an optimized selective-trapping and -delivery approach

The most important parameters in the system pertain to the conditions required for selective trapping/delivery of the VitD metabolites, which improves quantitative sensitivity and robustness by effectively removing hydrophobic/hydrophilic matrix components from samples. The performance of selective trapping/delivery was determined by two synchronized parameters: (i) the optimal organic mobile phase B% for high-flow-gradient selective trapping (B_trapping_%), and (ii) the optimal organic mobile phase B% of the analytical μLC gradient (B_analysis_%) for selective delivery of the VitD metabolites to the column; the trap was switched off the analytical column upon reaching the optimal B_analysis_%. Both parameters were experimentally identified.

The objective for optimization of B_trapping_% was to identify the maximal B_trapping_% that retained the VitD metabolites during the trapping process while maximizing the removal of more hydrophilic matrix components. As shown in Fig. 1b, the maximum B_trapping_% for 25(OH)D2, 25(OH)D3,1,25(OH)_2_D3 and 24,25(OH)_2_D3 were in the range of 32-35%. By balancing the considerations between efficient removal of hydrophilic components and the robustness of the procedure, a 31.5% B_trapping_% was determined optimal for selective trapping. The optimization of B_analysis_% was to identify the minimal B_analysis_% that delivered all four VitD metabolites to the analytical column while retaining more hydrophobic matrix components on the trap. It was observed that all four metabolites were completely transferred to the μLC analytical column when the trap was switched offline at a B_analysis_% of 60.5% (Fig. 1c**)**. Consequently, the optimal B_analysis_% to switch the trap off the analytical loop was established at 61.5%. The optimized gradient conditions and the time points for switching the trap on and off the μLC-MS system are shown in Supplementary Table 2. After determining the optimal B_trapping_% and B_analysis_%, the μLC gradient was fine-tuned to ensure the separation of the target peak from the endogenous interfering peaks within a runtime of 9 mins. The retention time for 25(OH)D3, 25(OH)D2,1,25(OH)_2_D3 and 24,25(OH)_2_D3 were 7.44 min, 7.44 min, 6.22 min and 5.10 min, respectively.

This selective trapping/delivery approach effectively reduced the matrix components in the injected samples, leading to reduced chemical noise and improved sensitivity for analyzing the four VitD metabolites. Supplementary Fig. 1 shows the comparison of the S/N with and without the optimized selective trapping/delivery strategy (*i.e.,* 31.5% B_trapping_% and 61.5% B_analysis_%). Clearly, the selective trapping/delivery strategy resulted in a 1.9–6.1fold increase in the S/N for the analysis of the four VitD metabolites.

#### S/N improvement by the narrow-window-isolation (NWI)-SRM

**Figure 2:**
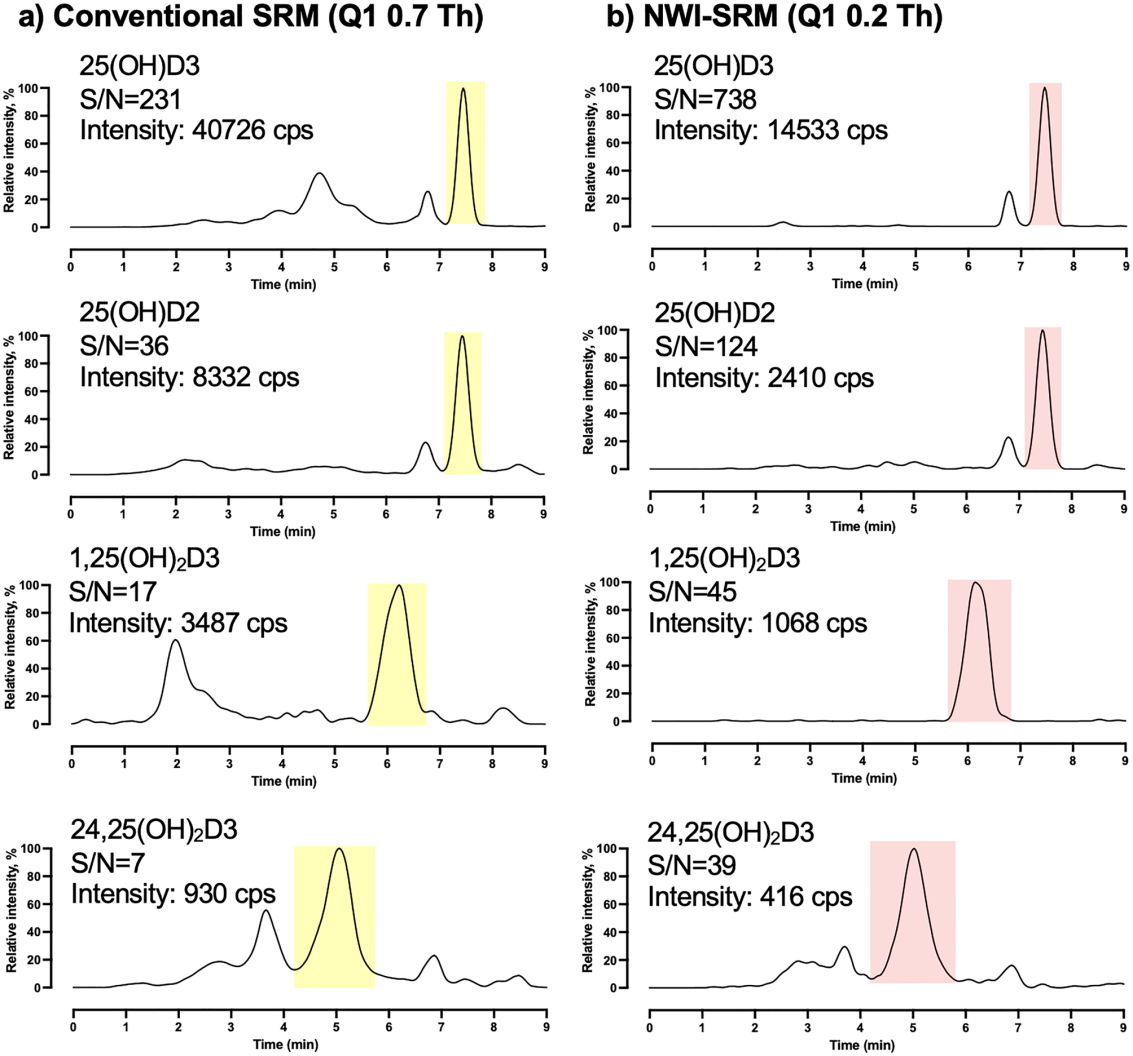
Comparison of the chromatograms of the four Vitamin D metabolites using (**a**) conventional Selected Reaction Monitoring (SRM) with Q1 0.7 Th and (**b**) Narrow-Window-Isolation (NWI)-SRM with Q1 0.2 Th in a pooled human serum sample. Signal-to-noise (S/N) ratios and absolute peak intensities in counts per second (cps) are marked for each metabolite.

The NWI-SRM utilizes a narrower *m/z* window for isolation of precursors at Q1 (*i.e.*, the first quadrupole), which effectively reduces the co-isolated species with close *m/z*, and thereby could substantially lower the chemical noise with a typically mild loss of signal intensity^29^. We have previously reported that NWI-SRM at a 0.2 Th resolution at Q1 enhances the S/N for quantification of proteins in biological samples over SRM with a conventional 0.7 Th window^29^. Here, we demonstrated that the NWI-SRM strategy markedly improved the sensitivity for quantification of VitD metabolites. Figure 2 shows the representative chromatograms of 25(OH)D3, 25(OH)D2, 1,25(OH)_2_D3 and 24,25(OH)_2_D3 in a pooled human serum sample after derivatization, comparing the conventional SRM (0.7 Th Q1) vs. NWI-SRM (0.2 Th Q1). When employing a 0.2 Th Q1 window, the signal response of target analytes decreased by 50-70% compared to using a conventional 0.7 Th window. However, there was a much higher reduction in chemical noise (>90%), leading to overall improvements in signal-to-noise ratio. As shown in Fig. 2, the NWI-SRM improved the S/N by 2.1-5.6 folds for the four VitD metabolites.

#### Further improvement of sensitivity by high-capacity sample loading on the large-I.D. trap

**Figure 3:**
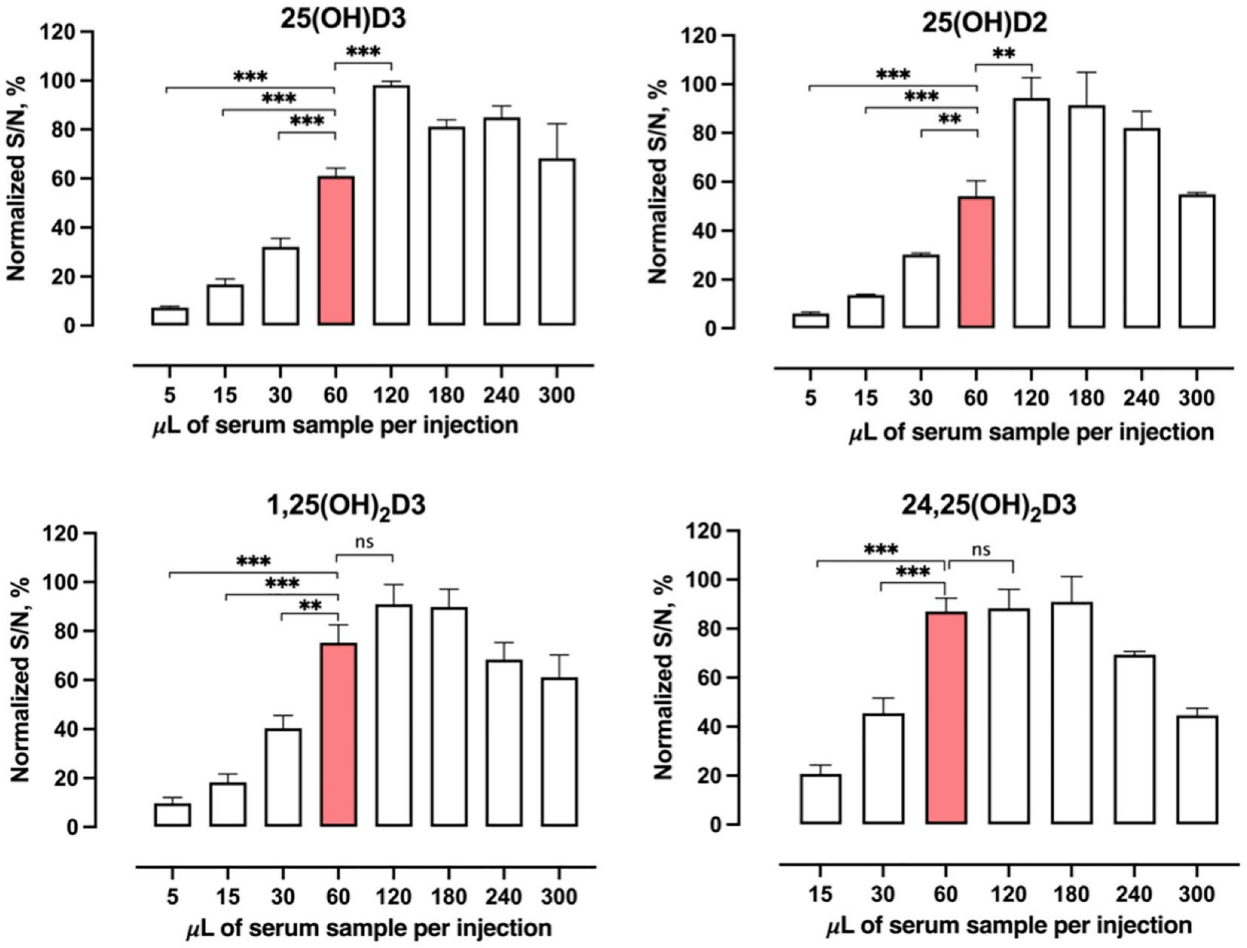
Enhanced sensitivity of four VitD metabolites achieved through high-capacity loading. Samples (i.e., reconstituted PTAD-derivatized serum extracts after protein precipitation, see “Method”) equivalent to varying volumes of pooled human serum sample were injected. The signal-to-noise (S/N) ratio of each metabolite at each injection volume was measured in 4 replicates using the optimized trapping-μLC-NWI-SRM method. The x-axis refers to the serum volume corresponding to the actual injected volume. The optimal injection volume (15 μL, corresponding to 60 μL serum) is marked in red. Two-tailed Student’s t-test *p*-value<0.01: **; *p*-value<0.001: ***; *ns*: no significance.

Given the effectively minimized chemical noises by the selective trapping/delivery and NWI-SRM approaches, an approach to enhance signal intensities would further increase the S/N of VitD metabolites. We hypothesized a high-capacity loading of samples, facilitated by the large-I.D. trap, may lead to markedly enhanced sensitivity for the VitD metabolites. To test this hypothesis, we investigated the S/N of the VitD metabolites as a function of loading amounts of samples. For all metabolites, the S/N was found to increase linearly along with the increased loading volumes in the range of 1.25-15 μL per injection, which corresponded to 5-60 μL serum sample per injection (Fig. 3). However, when the injection volume increased to 30 µL, which corresponded to 120 µL serum, the S/N of 25(OH)D3 or 25(OH)D2 continued to rise, while no significant increase in S/N was observed for 1,25(OH)_2_D3 or 24,25(OH)_2_D3. We speculate that this is because the loading capacity of the more hydrophilic analytes such as 1,25(OH)_2_D3 and 24,25(OH)_2_D3 have been reached at this volume, for reasons specified previously^29^. By balancing the considerations of sensitivity gains and serum consumption, the injection volume 15 μL, which corresponded to 60-μL serum loading per analysis, was selected. Such high-capacity loading further enhanced sensitivity by 2-4 folds compared to the typical loading capacity in a conventional μLC-MS (*i.e.*, injection volumes capped at a volume equal to 15-30 μL serum sample).

#### Evaluation of method robustness

For large-cohort clinical analysis, maintaining a high method robustness is crucial. Conventional μLC-MS often experiences markedly decreased operational robustness because of the accumulation of detrimental matrix components on the spray needle, leading to severe signal drop and the need of instrument cleaning after the injection of a limited amount of plasma samples^29^. By comparison, the selective-trapping and delivery approach efficiently prevents these detrimental matrix components from entering the μLC-MS system while selectively delivering target compounds into the μLC-MS system, boosting the operational robustness. To evaluate the robustness of the T-μLC-MS system, we monitored the signal intensity of a processed pooled human serum sample injected every ~15 runs of project samples. The signal intensity of each VitD metabolite in the processed pooled human serum sample remained within ±25% of these from the first injection (Supplementary Fig. 2). Therefore, the developed T-μLC-MS method can continuously quantify an extensive cohort (i.e., at least 600 samples) of biological samples without the need to interrupt the runs, indicating excellent robustness.

#### Evaluation of the overall quantitative sensitivity and method validation

We performed a comprehensive validation on the stability of analytes, as well as the selectivity, sensitivity, accuracy and precision of the developed method.

Stability was tested on several conditions, including multiple freeze-thaw cycles from −80 to 22 °C, storage at 4 °C, and storage at ambient temperature. Both 25(OH)D2 and 25(OH)D3 were found to be highly stable in these conditions, which agreed with previous reports^27,35^. In line with our previous study^27^, underivatized 1,25(OH)_2_D3 was stable at 4 °C over 2 h and at −80 °C for 60 days. Underivatized 1,25(OH)_2_D3 was found stable for 3 freeze-thaw cycles, and its derivatized form was stable in a cooled autosampler (4 °C) for 96 h. The 24,25(OH)_2_D3 was found stable under the above conditions.

Selectivity was evaluated by testing surrogate matrix from six preparations to determine whether endogenous peaks interfered at the SRM transitions chosen for each metabolite or I.S.. Supplementary Fig. 3 shows the representative chromatogram of a blank surrogate matrix. No interference at the retention times of each metabolite or I.S. was observed. The selectivity of each metabolite and I.S. in pooled human serum was confirmed by comparing the ratio of peak area in two transitions for each metabolite or I.S. under multiple reaction monitoring (MRM) mode in the established 9-min method versus the ratio obtained with a 45-min extensive gradient separation. No additional peaks was separated by extensitve gradient separation (Supplementary Fig. 4), and the ratio of peak area in two MRM transitions was found to be consistent between 9-min method and extensive gradient separation (Supplementary Fig. 5), demonstrating the high selectivity in the pooled human serum.

**Figure 4:**
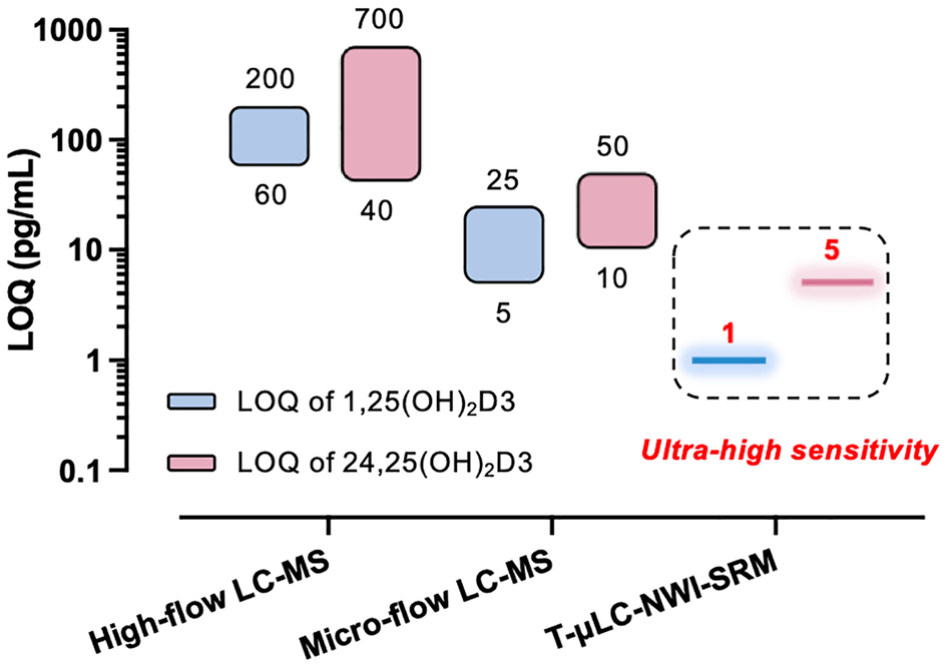
Ultra-high sensitivity of the T-μLC-NWI-SRM method in comparison to published methods utilizing either high-flow LC-MS (flow rate > 200 μL/min) or micro-flow LC-MS (flow rate between 1 and 200 μL/min) for quantification of two di-hydroxy VitD metabolites. The numbers above and below the boxes represent the range of reported Limit of Quantifications (LOQs) in literature.

By incorporating the selective trapping/delivery, NWI-SRM and high-capacity loading, the developed strategy showed exceptional quantitative sensitivity. Within a 9-min analytical cycle, the method achieved LOQs of 1.0 pg/mL for 1,25(OH)_2_D3, 5.0 pg/mL for 24,25(OH)_2_D3, 30 pg/mL for both 25(OH)D2 and 25(OH)D3 (Table 1). By comparison, in existing methods, LOQs of 60-200 pg/mL for 1,25(OH)_2_D3 and 40-700 pg/mL for 24,25(OH)_2_D3 have been achieved by high-flow LC-MS, and LOQs of 60-200 pg/mL for 1,25(OH)_2_D3 and 40–700 pg/mL for 24,25(OH)_2_D3 by μLC-MS^3,27,36–42^. As illustrated in Fig. 4, the LOQs achieved by T-µLC-NWI-SRM are superior to existing methods using either high-flow-LC-MS or μLC-MS. Method accuracy and precision were evaluated using quality control (QC) samples prepared by spiking standard compounds into both surrogate matrix (referred as the “blank QC”) and a pooled human serum sample (referred as the “fortified QC”) for which the endogenous levels of the analytes were previously measured. Table 2 shows that, at all QC levels, good accuracies with relative error <15% were obtained for all targeted VitD metabolites, and the quantitative errors were within ±15% of the nominal concentration. No appreciable matrix effect was observed.

**Table 1:** Sensitivity, and linearity range for the quantification of the four metabolites.

VitD metabolites	1,25(OH)_2_D3	24,25(OH)_2_D3	25(OH)D3	25(OH)D2
LOD* pg/mL	0.2	0.5	15	15
LOQ pg/mL	1.0	5.0	30	30
Linear range (pg/mL)	1–800	5–40,000	30–60,000	30-60,000

*Defined as S/N=3.

**Table 2:** The validation data for quantification of the four VitD metabolites in serum.

VitD metabolites	Nominal concentration (pg/mL)	Intra-day (n=6)			Inter-day (n=6)		
		Calculated concentration [Mean ± SD, (pg/mL)]	Relative error (%)	% CV	Calculated concentration [Mean ± SD, (pg/mL)]	Relative error (%)	% CV
Blank QCs^a^							
1,25(OH)_2_D3	1.0	0.95 ± 0.05	− 5	5.3	1.12 ± 0.07	12	6.3
	6.0	5.9 ± 0.3	− 2	4.6	6.2 ± 0.5	3	8.0
	40.0	42.6 ± 4.5	7	10.6	38.5 ± 4.4	− 4	11.0
	200.0	206.0 ± 7.9	3	3.9	204.7 ± 10.9	2	8.7
24,25(OH)_2_D3	5.0	5.2 ± 0.5	4	9.6	4.9 ± 0.6	−2	11.2
	30.0	31.4 ± 3.1	5	10.0	30.7 ± 1.9	2	6.4
	200.0	209.2 ± 11.0	5	5.3	218.2 ± 10.9	9	5.0
	1000.0	1046.9 ± 70.2	3	7.2	1044.2± 75.1	4	7.2
25(OH)D3	30.0	31.2 ± 2.8	4	8.8	28.9 ± 3.9	− 4	13.4
	1000.0	1020.0 ± 83.1	2	8.1	960.0 ± 63.4	2	6.6
	6670.0	6620.2 ± 290.3	− 1	4.4	6280.7 ± 500.1	− 6	8.0
	33300.0	33605.3 ± 1190.1	1	3.5	32823.6 ± 515.6	− 1	1.6
25(OH)D2	30.0	30.7 ± 3.6	2	11.6	29.8 ± 3.0	− 1	10.0
	200.0	190.7 ± 8.8	− 6	4.6	183.5 ± 13.8	− 8	7.5
	1330.0	1244.1 ± 93.9	− 6	7.5	1271.7 ± 64.4	− 5	5.1
	6670.0	6412.6 ± 210.2	− 4	3.3	6152.9 ± 229.8	− 8	3.7
Fortified QCs^b,c^							
1,25(OH)_2_D3	16.7	15.2 ± 0.9	− 9	5.6	15.5 ± 0.6	− 7	4.1
	50.0	48.5 ± 2.3	− 3	4.7	46.5 ± 0.3	− 7	0.7
24,25(OH)_2_D3	250.0	275.0 ± 1.7	10	0.6	272.5 ± 22.1	9	8.1
	750.0	832.5 ± 44.1	11	5.3	742.5 ± 84.6	− 1	11.4
25(OH)D3	1670.0	1603.2 ± 89.8	− 4	5.6	1586.5 ± 128.5	− 5	8.1
	5000.0	4600.0 ± 317.4	− 8	6.9	5150.0 ± 170.0	3	3.3
25(OH)D2	1670.0	1569.8 ± 168.0	− 6	10.7	1653.3 ± 77.7	− 1	4.7
	5000.0	4900.0 ± 137.3	− 2	2.8	4750.0 ± 427.5	− 5	9.0

^a^Prepared by spiking standard compounds into blank matrix.

^b^Prepared by spiking standard compounds into a pooled human serum.

^c^The endogenous level has been subtracted from the measured value.

Taking together, the validation results showed that the T-μLC-NWI-SRM method achieved excellent quantitative selectivity, sensitivity, accuracy, precision as well as high throughput.

### Application of T-μLC-NWI-SRM method for quantification of VitD metabolites in multiple sclerosis patients

We employed the developed T-μLC-NWI-SRM method to analyze serum samples from 218 multiple sclerosis patients. The demographic and clinical characteristics of the multiple sclerosis patient cohort are described in Supplementary Table 3. As shown in Supplementary Fig. 6, the LOQ achieved by T-μLC-NWI-SRM method were below the levels of the four VitD metabolites in the majority of the cohort, enabling a comprehensive investigation of VitD metabolites in the multiple sclerosis patients. Table 3 shows that, in the multiple sclerosis patient cohort, the average total 25(OH)D level was 32.68 ng/mL, and the concentrations of 25(OH)D3 and 25(OH)D2 were 25.63±12.17 ng/mL and 7.04±14.41 ng/mL (mean±SD, same below) respectively. The concentrations of 1,25(OH)_2_D3 and 24,25(OH)_2_D3 were 0.027±0.018 ng/mL and 1.02±0.89 ng/mL respectively. Among these patients, 40% (41/218) had serum 25(OH)D2 levels higher than the published baseline 25(OH)D2 level, (i.e., <5 nmol/L or 2.06 ng/mL^43^).

**Table 3:** The average and median concentrations of each VitD metabolite in the multiple sclerosis patient cohort.

	Mean (ng/mL)	SD (ng/mL)	Median (ng/mL)
1,25(OH)_2_D3	0.027	0.018	0.025
24,25(OH)_2_D3	1.02	0.89	0.81
25(OH)D3	25.63	12.17	25.51
25(OH)D2	7.04	14.41	1.17
Total 25(OH)D*	32.68	13.56	30.83

*Total 25(OH)D is calculated as the sum of 25(OH)D3 and 25(OH)D2 for each patient.

### The observed negative correlation between the levels of 25(OH)D2 and the levels of three D3 metabolites in multiple sclerosis patients

Numerous studies have established a link between the low levels of circulating 25(OH)D and an increased risk and prevalence of multiple sclerosis^44^, with levels tending to decrease further in later stages of the disease^45^. Therefore, individuals with multiple sclerosis are suggested to take VitD supplements to maintain circulating 25(OH)D levels^34^. While both D2 and D3 forms are available as VitD supplements, studies found that their metabolites have different pharmacokinetic properties and biological effects^15–18^. Moreover, it was speculated that D2 may impact D3 metabolism^6^. For example, previous studies in healthy population and patients with chronic kidney disease have suggested a negative correlation between circulating levels of 25(OH)D3 and 25(OH)D2, in either observational or D2 intervention studies^46,47^.

Using the technique developed here, we examined whether such a negative correlation between 25(OH)D2 and 25(OH)D3 may also be present in multiple sclerosis patients. Furthermore, 25(OH)D3 is metabolized into 1,25(OH)_2_D3 (Supplementary Fig. 7), which exerts immunomodulatory effects and regulates neurogenesis and neuroprotection, potentially contributing to the potential protective effects of D3 in multiple sclerosis^48^. The 25(OH)D3 is also metabolized into 24,25(OH)_2_D3 (Supplementary Fig. 7), which was reported to show a strong association with disability scores of multiple sclerosis patients^28^. Thus, this study also aims to investigate whether these di-hydroxyl D3 metabolites correlate with 25(OH)D2 in multiple sclerosis patients, a topic that has not been previously explored.

**Figure 5:**
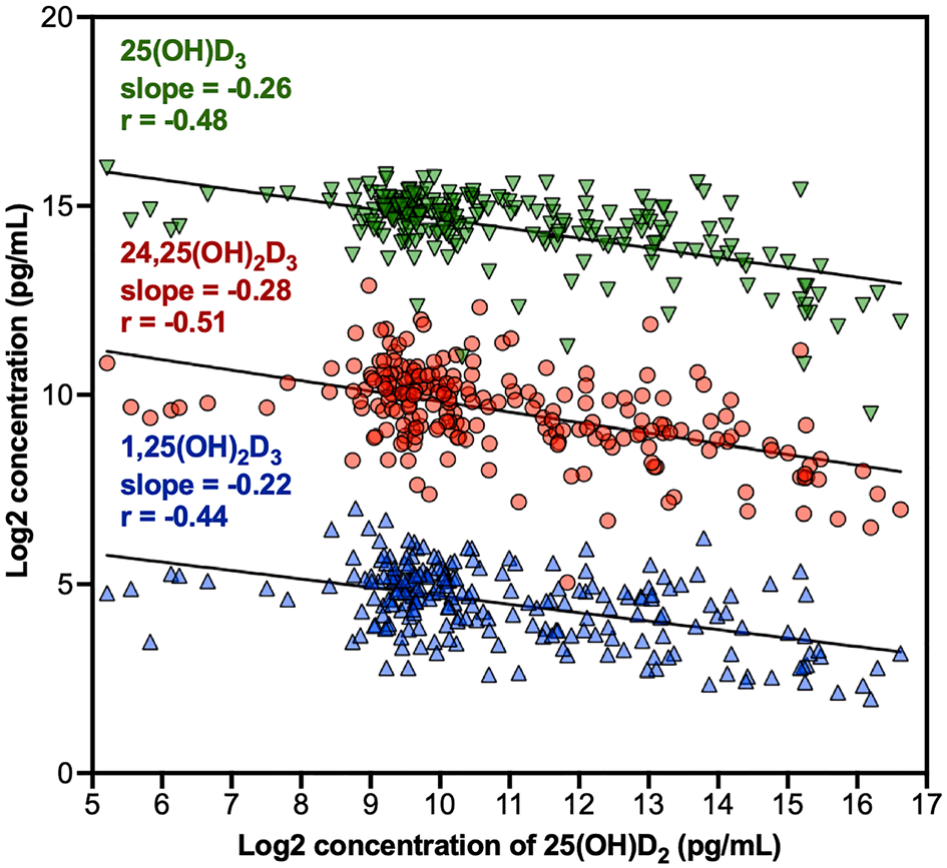
The negative correlation between the levels of 25(OH)D_2_ and the levels of 25(OH)_2_D_3_, 24,25(OH)_2_D_3_, and 25(OH)D_3_ in the serum samples of 218 multiple sclerosis patients.

Using the quantitative data from the cohort of multiple sclerosis patients, we conducted nonparametric Spearman correlation analysis and calculated the linear regression slopes between 25(OH)D2 and each D3 metabolite. The results showed negative correlations of the levels of 25(OH)D2 and the levels of 25(OH)D3 (slope=− 0.26, r=− 0.48, *p*-value<0.0001) in multiple sclerosis patients, consistent with previous studies in non-multiple-sclerosis populations that demonstrated greater D2 intake being associated with lower D3 metabolite levels^6,12,13,46,47,49–51^. Moreover, we also observed apparent negative correlations between the levels of 25(OH)D2 and both 1,25(OH)_2_D3 (slope=− 0.22, r=− 0.44, *p*-value<0.0001) and 24,25(OH)_2_D3 (slope=− 0.28, r=− 0.51, *p*-value<0.0001, Fig. 5). The negative correlation observed between 25(OH)D2 and the D3 metabolites may result from competition for the shared metabolic pathways between D2 and D3, or from altered elimination pathways for VitD metabolites in individuals with multiple sclerosis. Further investigation is warranted to elucidate the underlying mechanisms.

## Discussion

Here we developed a T-µLC-NWI-SRM method to address the growing demand for sensitive, high-throughput, and robust methods to measure multiple VitD metabolites in clinical samples. Despite that high-flow LC-MS (i.e., flow rate > 200 μL/min) is commonly employed for quantification of VitD metabolites in order to achieve a high-throughput analysis^37,39,40,52^, they often lacks sufficient sensitivity for the di-hydroxyl VitD metabolites. On the other hand, micro-flow LC-MS (i.e., typical flow rates at 10-50 μL/min) provides higher sensitivity, but is frequently limited by low robustness, low throughput, and the requirement of additional sample preparation procedures to maintain operational robustness^27,41,53^. The T-µLC-NWI-SRM method has addressed these challenges by: (i) employing a selective-trapping and delivery approach to efficiently remove matrix components, enabling high-capacity sample loading and enhancing sensitivity, throughput, and robustness; (ii) implementing the NWI-SRM strategy, which further improves sensitivity by providing high selectivity. Taken together, the T-µLC-NWI- SRM achieved LOQs that were markedly lower than any existing LC-MS methods, within a 9-minute short analytical cycle and with exceptional robustness across large cohort clinical samples.

Compared to the existing LC-MS-based methods^3,27,36–42^, the T-μLC-NWI-SRM strategy developed here achieves drastically lower LOQ for the two di-hydroxyl VitD metabolites (Fig. 4). This ultra-high sensitivity is accomplished through the combination of high-capacity sample loading and selective trapping/delivery on the large I.D. trap, as well as the selective NWI-SRM detection. The T-μLC-NWI-SRM strategy also enhances throughput by providing online sample clean-up, eliminating the needs for solid-phase extraction; additionally, the selective trapping/delivery approach eliminates most of the matrix components so that μLC-MS system can focus on the analysis of the target VitD metabolites, which enables a short analytical cycle of only 9 min. Finally, the selective trapping/delivery approach also enhances method robustness by preventing detrimental matrix components from entering the µLC-MS system, as evidenced by the absence of perceivable signal loss after over 600 injections of biological samples (Supplementary Fig. 2). Overall, this strategy enables ultra-sensitive, high-throughput, and robust quantification of multiple D2/D3 metabolites in serum samples.

Previous studies reported declined 25(OH)D3 concentrations in both healthy individuals and patients with chronic kidney diseases taking D2 supplementation^6,12,46,47^. However, the relationship between 25(OH)D2 and the di-hydroxylated VitD metabolites that are critical to the functions of VitD, has been rarely investigated, likely due to technical limitations in sensitive quantification of these metabolites^6,46,47^. Furthermore, while previous research has suggested a potential interplay between multiple sclerosis and various VitD metabolites, the levels of di-hydroxyl VitD metabolites have not been adequately surveyed in multiple sclerosis patients^54^. Here, we measured VitD metabolites in over 200 multiple sclerosis patients and observed a negative correlation between 25(OH)D2 levels and the levels of D3 metabolites, including 25(OH)D3, 1,25(OH)_2_D3, and 24,25(OH)_2_D3 in the patient cohorts. The observation may be attributed to the competitive binding of D2 to the saturable VitD 25-hydroxylase enzyme shared by D2 and D3^55^, leading to decreased 25(OH)D3 levels. As 25(OH)D3 is a precursor to both 1,25(OH)2D3 and 24,25(OH)2D3^56,57^, the concentrations of these di-hydroxylated metabolites may also be negatively impacted by D2 ingestion. This observation could prove valuable in managing multiple sclerosis. One limitation of our data interpretation is the lack of information on the type and dosage of VitD supplements in the patient cohort. However, our observational results are consistent with previous interventional studies that reveals the negative association between 25(OH)D2 and 25(OH)D3^6,12,46,47^.

In summary, we established T-μLC-NWI-SRM strategy allowing sensitive, high-throughput and robust measurement of multiple VitD metabolites in serum samples pretreated by derivatization, which is valuable in large-cohort clinical studies. By applying this method to a large-cohort of serum samples from multiple sclerosis patients, we gained valuable insights into VitD metabolism in multiple sclerosis patients, which facilitates improved treatment and management of the disease.

## Method

### Chemicals and reagents

The standard of 25(OH)D_2_ was purchased from Sigma Aldrich (St. Louis, MO). Standards of 25(OH)D_3_ and 1,25(OH)_2_D_3_ were obtained from MilliporeSigma (Burlington, MA). The standard of 24,25(OH)_2_D_3_ was from MP Biomedicals (Irvine, CA). Stable isotope labeled internal standards (I.S.) (*i.e., d*_*6*_-1,25(OH)_2_D_3_, *d*_*6*_-25(OH)D_2_ and *d*_*6*_-25(OH)D_3_) were purchased from Medical Isotope (Pelham, NH). 4-phenyl-1,2,4-triazoline-3,5-dione (PTAD), used as a derivatization reagent, was obtained from Sigma Aldrich (St. Louis, MO), along with bovine serum albumin (BSA). Phosphate buffered saline (PBS) and HPLC grade acetonitrile (ACN) were purchased from Fisher Chemicals (Waltham, MA). Water was deionized by a Milli-Q water purification system (MilliporeSigma, MA). Formic acid (FA) was purchased from Sigma-Aldrich (St. Louis, MO).

### Study population

The study enrolled 218 adult patients with clinically definite multiple sclerosis in Jacobs Multiple Sclerosis Center for Treatment and Research, University at Buffalo. Of the patients,184 were diagnosed with relapsing-remitting multiple sclerosis (RRMS) patients and 34 with progressive multiple sclerosis (PMS), which included both secondary progressive multiple sclerosis (SPMS) and primary progressive MS (PPMS). The demographic characteristics of the multiple sclerosis patient cohort is shown in Supplementary Table 3. The study was conducted in accordance with the Declaration of Helsinki, and the protocol was approved the University at Buffalo Health Sciences Institutional Review Board. Written informed consent was obtained from all patients at enrollment.

### Serum sample preparation

Serum samples were stored at – 80 °C in polypropylene micro-centrifuge tubes until been processed. Once thawed, the serum samples were vortexed, and 200 µL of each serum sample was transferred into a new tube, spiked with 5 µL of an I.S. stock solution containing 36 ng/mL *d*_*6*_-1,25(OH)_2_D_3_, 216 ng/mL *d*_*6*_-25(OH)D_2_ and 216 ng/mL *d*_*6*_-25(OH)D_3_ in ACN. The samples were placed at 4 °C to equilibrate for 30 min. Proteins were precipitated by adding 1 mL MeOH, followed by rigorous vortex for 2 min. After centrifugation at 20,000 *g* for 20 min at 4 °C, the supernatants were transferred to another tube and evaporated under nitrogen stream until completely dried. 200 µL of 1 mg/mL PTAD in ACN was added into the residue for derivatization of VitD metabolites. The reaction was carried out at room temperature for 2 h and terminated by adding 800 µL water. The terminated reaction mixture was dried under nitrogen stream with 37 °C water bath, and then reconstituted with 50 µL of 30% ACN containing 1% FA. The scheme for derivatization reaction is shown in Supplementary Fig. 8.

### Trapping-micro-LC-narrow window isolation-selective reaction monitoring (T-μLC-NWI-SRM)

An UltiMate 3000 LC system (containing an SRD-3400 degasser, NCS-3500RS CAP pumps, a high-flow tertiary gradient pump, and a WPS-3000TBRS autosampler with a 250 μL loop) coupled to a TSQ Quantiva triple-quadrupole mass spectrometer via an Ion Max NG ion source with an H-ESI probe and a 34-G narrow-bore spray needle (Thermo Fisher Scientific, CA) was used. Sample trapping was conducted on a C8 trapping column (15 × 2.1 mm, 3.5 μm particle size, 100 Å, Agilent, CA) at a flow rate of 1 mL/min using the tertiary pump. The high-flow loading mobile phases (MPs) A_trapping_ and B_trapping_ were water:ACN:FA 98:2:0.1 (v/v/v, pH 3.0) and water:ACN:FA 5:95:0.1 (v/v/v, pH 3.0), respectively. A micro-flow selector (5-50 μL/min) was used for μLC-MS. The separation column was an in-house packed column (150 × 0.5 mm, 2.2 µm, 120Å) using the packing material of Acclaim™ 120 C18 column (Thermo Fisher, MA) at a flow rate of 25 μL/min. The micro-flow mobile phases A_analysis_ and B_analysis_ were water:ACN:FA 98:2:0.1 (v/v/v, pH 3.0) and water:ACN:FA 5:95:0.1 (v/v/v, pH 3.0), respectively. A ZDV six-port valve placed in the heated column compartment was utilized to coordinate the operations of the two flow systems, with the separation temperature controlled at 40 °C. At the beginning of the sample delivery from the trap to the column, a 1-min isocratic elution with the initial gradient B_analysis_ percentage was used to for peak compression. The LC gradient for the T-μLC system is shown in Supplementary Table 2. The total run time was 9 min. The MS instrument parameters were set as following: spray voltage was at 3.5 kV, the vaporizer temperature at 50 °C, the sheath gas at 8 arb unit, the auxiliary gas at 6 arb unit, and the capillary temperature was maintained at 325 °C. The optimized RF Lens voltages and collision energies were obtained for each target analyte by an on-the-fly orthogonal-array-optimization (OAO) described in our previous publication^29^. The isolation window for NWI-SRM was set to 0.2 Th for Q1 and 0.7 Th for Q3 for all channels. The MS transitions of each target are shown in Supplementary Table 1.

### Optimization of the T-μLC conditions

A pooled human serum sample was prepared by taking an equal volume of each serum sample from 25 randomly-selected multiple sclerosis patient serums. The pooled human serum sample was processed using the procedures described in the serum sample preparation section. The sample was then used for T-μLC conditions optimization. To determine the optimal B_trapping_%, series of different loading conditions (B_trapping_% ranging from 10 to 50%) in a step size of 5% were evaluated with a generic delivery condition (*i.e.,* the trap was not switched offline until the end of analysis) to the analytical column. The trapping column was equilibrated with the next experimental condition at the end of each analysis cycle to prepare for the next analytical cycle. To determine the optimal B_analysis_%, similarly, a series of different delivery conditions (*i.e.*, the B_analysis_% during the gradient elution to switch the trap off the column) was evaluated in a step size of 1.5% for the trap-switching B_analysis_% ranging from 50 to 85%, with a mild loading condition (B_trapping_% = 1%). Finally, the analytical gradient was finetuned to ensure the separation of the target peak from the endogenous interfering peaks in the shortest run time. To evaluate the sensitivity improvement of four VitD metabolites by high capacity loading, the sample prepared with a pooled human serum was injected at different volumes which equals to 5, 15, 30, 60, 120, 180, 240 or 300 μL serum, and each volume was injected 4 times.

### Comparison between signal-to-noise (S/N) ratio of with and without selective-trapping/delivery

A pooled human serum sample was experimentally prepared by combining an equal volume of serum from each of the 25 randomly selected multiple sclerosis patients. The conditions for selective trapping/delivery are described above. To measure S/N ratio of each metabolite without selective trapping/delivery, the same sample was injected, and the T-μLC method was as follows: during sample loading (0–0.3 min), the B_trapping_% was set to 1% so that the vast majority of the sample components were retained on the trapping column; after switching the trapping column in line with the analytical column at 0.4 min, the trapping column remained in-line till the end of separation cycle, so that all trapped compounds were delivered to the analytical column. Samples were analysis in triplicate for either condition.

### Calibration and assay validation

An I.S. stock solution was prepared in ACN containing 36 ng/mL *d*_*6*_-1,25(OH)_2_D_3_, 216 ng/mL *d*_*6*_-25(OH)D_2_ and 216 ng/mL *d*_*6*_-25(OH)D_3_. *d6*-1,25(OH)_2_D_3_ was used as the I.S. for both 1,25(OH)_2_D_3_ and 24,25(OH)_2_D_3_. Calibration standards were prepared at six levels across the linear range (Table 1) with three replicates at each level by spiking known amounts of VitD metabolites into a surrogate matrix comprised of 50 mg/mL BSA in PBS.

For stability investigation, 20 mL of pooled serum from healthy subjects was spiked with 1 ng/mL or 10 ng/mL of each metabolite, respectively. Aliquots of samples were subjected to various evaluations including multiple freeze-thaw cycles (from − 80 to 22 °C), storage at ambient temperature and storage at 4 °C. At each designated intervals, isotope-labeled I.S. was added, and then the sample were stored at − 80 °C until analysis. Samples was processed using the procedures described above and analzyed with the optimized T-μLC-NWI-SRM method.

For the evaluation of accuracy and precision, blank quality control (QC) samples were prepared by spiking VitD metabolites into the surrogate matrix at four concentration levels including LOQ, and fortified QCs were prepared in a pooled human serum at two levels (Table 2). All QC samples were prepared in six replicates at each QC level. Calibration standards and QC samples were processed using the procedures described above. The optimized T-μLC-NWI-SRM method was used for analysis of calibration standards and QC samples. The data was processed using Skyline v22.2. Calibration curves were constructed by plotting the ratio of peak area of each metabolite to the peak area of corresponding I.S. at each concentration level, and then fitting the data using weighted least-squares linear regression with a 1/X^2^ weighting factor. Accuracy and precision were evaluated intra-day on the same day and inter-day on three consecutive days. Accuracy was calculated as the relative error percent of the average concentration relative to the nominal concentration of each QC level. Precision was calculated as the coefficient of variation of the measured concentrations of six replicates of QC samples (Table 2).

For selectivity evaluation in surrogate matrix, BSA from six preparations were tested. Surrogate matrix was processed using the procedures described above and analzyed with the optimized T-μLC-NWI-SRM method. Selectivity in human serum was tested with pooled human serum sample by mixing an equal volume of each serum sample from 25 randomly-selected multiple sclerosis patient serums. The pooled sample was processed using the procedures described above. A modified T-μLC-NWI-SRM method was used to analyzed the processed sample to enable an 45-min extensive separation gradient on µLC.

Method robustness was evaluated by injecting the pooled human serum sample every ~15 injections of others samples. The response ratio to the first analysis of each metabolite was calculated by dividing the peak intensity in each analysis to the peak intensity in the first analysis.

### Supplementary Information

Supplementary Information.

## Data Availability

The mass spectrometric datasets are available from the corresponding author (X.Z.) on reasonable request. The clinical data are not publicly available due to privacy and ethical restrictions.
