# Supplementary material for: An ultra-sensitive and high-throughput trapping-micro-LC-MS method for quantification of circulating vitamin D metabolites and application in multiple sclerosis patients
Source: Sci Rep. 2024 Mar 6;14:5545. doi: 10.1038/s41598-024-55939-0 (PMC10918069; doi:10.1038/s41598-024-55939-0)
Supplement: Supplementary file 1 — Supplementary Information. [file 41598_2024_55939_MOESM1_ESM.docx]

**An Ultra-Sensitive and High-Throughput Trapping-micro-LC-MS Method for Quantification of Circulating Vitamin D Metabolites and Application in Multiple Sclerosis Patients**

Flora Qu^1,2^, Ming Zhang^2^, Bianca Weinstock-Guttman^3^, Robert Zivadinov^4,5^, Jun Qu^2,6,^, Xiaoyu Zhu^2,*^, Murali Ramanathan^2,*^

1 Department of Biochemistry, University at Buffalo, State University of New York, Buffalo, NY, USA

2 Department of Pharmaceutical Sciences, University at Buffalo, State University of New York, Buffalo, NY, USA

3 Department of Neurology, Jacobs Comprehensive MS Treatment and Research Center, Jacobs School of Medicine and Biomedical Sciences, University at Buffalo, State University of New York, Buffalo, NY, USA

4 Buffalo Neuroimaging Analysis Center, Department of Neurology, Jacobs School of Medicine and Biomedical Sciences, University at Buffalo, State University of New York, Buffalo, NY, USA;

5 Center for Biomedical Imaging at the Clinical Translational Science Institute, University at Buffalo, State University of New York, Buffalo, NY, USA

6 New York State Center of Excellence in Bioinformatics & Life Sciences, Buffalo, NY, USA

*Corresponding authors

**Xiaoyu Zhu**

E-mail: [xzhu27@buffalo.edu](mailto:xzhu27@buffalo.edu)

**Murali Ramanathan**

E-mail: murali@buffalo.edu

**Supplementary Table 1**. MS transitions of targets and internal standards

|  | **Precursor *m/z*** | **Product *m/z*** | **Collision Energy (eV)** | **RF lens (V)** |
| --- | --- | --- | --- | --- |
| **25(OH)D3** | 558.42 | 298.1 | 19 | 119 |
| **25(OH)D3-*d6*** | 564.35 | 298.3 | 19 | 119 |
| **25(OH)D2** | 570.15 | 298.1 | 18 | 96 |
| **25(OH)D2-*d6*** | 576.27 | 298.3 | 18 | 96 |
| **24,25(OH)_2_D3** | 574.31 | 298.2 | 20 | 104 |
| **1,25(OH)_2_D3** | 574.41 | 314.1 | 19 | 128 |
| **1,25(OH)_2_D3-*d6*** | 580.43 | 314.0 | 19 | 128 |

**Supplementary Table 2**. LC gradient for the trapping-micro-LC system consisting of a high-flow-trapping LC and a synchronized micro-flow-LC

| **LC system** | **Time (min)** | **Flow rate (µL/min)** | **High-flow LC mobile phase B (%)** | **Events/Comments** |
| --- | --- | --- | --- | --- |
| High-flow trapping LC | 0.0 | 1000 | 31.5 | Start sample loading onto trapping column. |
|  | 0.3 | 1000 | 31.5 | Switch the trapping column in line with the separation column, end sample trapping and start sample delivery onto separation column. |
|  | 0.4 | 300 | 31.5 | Deliver sample onto separation column. Switch high-flow pump off line with the trapping column. Adjust flow rate to decrease mobile phase usage. |
|  | 4.5 | 300 | 31.5 |  |
|  | 4.6 | 300 | 31.5 | Switch the trapping column off the separation column to end sample delivery onto separation column. |
|  | 4.7 | 1000 | 99.0 | Trapping column clean-up |
|  | 7.9 | 1000 | 99.0 |  |
|  | 8.0 | 1000 | 31.5 | Trapping column equilibration |
|  | 9.0 | 1000 | 31.5 |  |
| **LC system** | **Time (min)** | **Flow rate (µL/min)** | **Micro-flow LC mobile phase B (%)** | **Events/Comments** |
| Micro-flow-LC | 0.0 | 25 | 40.0 | Separation column equilibration |
|  | 0.3 | 25 | 40.0 |  |
|  | 1.3 | 25 | 40.0 | Peak compression |
|  | 4.6 | 25 | 61.5 | Separation column in line with trapping column. Start sample separation. |
|  | 4.7 | 25 | 48.4 |  |
|  | 7.4 | 25 | 66.0 |  |
|  | 7.5 | 25 | 99.0 | Separation column clean-up |
|  | 8.0 | 25 | 99.0 |  |
|  | 8.1 | 25 | 40.0 | Separation column equilibration |
|  | 9.0 | 25 | 40.0 |  |

**Supplementary Table 3** The demographic characteristics of the multiple sclerosis patient cohort

| **Characteristics** |  | **Value** |
| --- | --- | --- |
| n |  | 218 |
| Women: Men |  | 170:48 |
| Multiple sclerosis type |  |  |
| RP-MS |  | 184 |
| PMS |  | 34 |
| Age (years) |  | 45.0±11.7 |
| Disease duration (years) |  | 13.7±10.2 |
| MSSS |  | 3.4±2.3 |
| EDSS |  | 3.0±1.9 |
|  |  |  |

Data is mean±SD for age, disease duration, MSSS and EDSS.

RP-MS, relapsing-remitting multiple sclerosis; PMS, progressive multiple sclerosis; Multiple Sclerosis Severity Scale; EDSS, Expanded Disability Status.

**
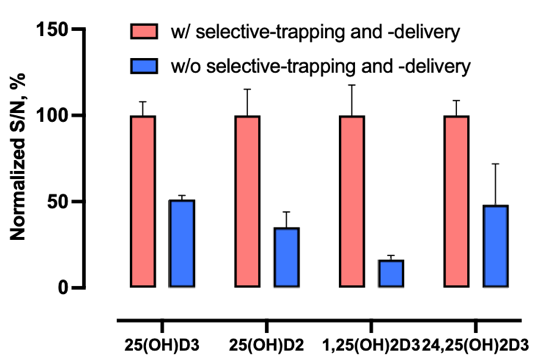
**

**Supplementary Figure 1** Increased signal-to-noise S/N (ratio) by the optimized selective-trapping and -delivery approach measured by pooled human serum. The sample was run in triplicates for each method.


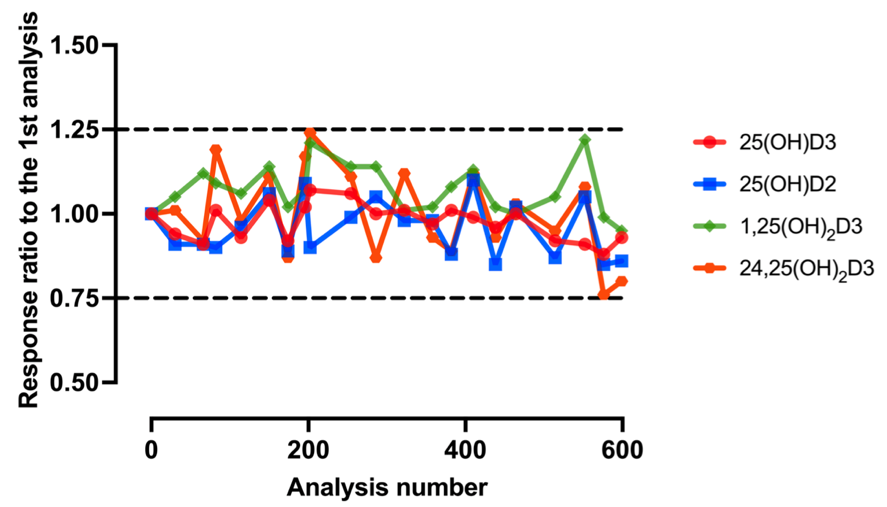


**Supplementary Figure 2** The high robustness of the T-μLC-MS system. Surrogate matrix spiked with the 4 metabolites were injected every ~15 injections of others samples. No appreciable decreases in the signals were observed after >600 injections.

**Supplementary Figure 3** Representative chromatogram of blank surrogate matrix. The SRM transitions for quantification of four Vitamin D metabolite are shown.

**Supplementary Figure 4** Representative chromatogram of pooled human serum with a 45-min extensive separation method. The SRM transitions for quantification of four Vitamin D metabolite are shown. No interference peak was separated.

**Supplementary Figure 5** Ratio of peak area in two transitions under multiple reaction monitoring (MRM) mode for Vitamin D metabolites and I.S.. The ratios were the same between target and I.S., and were consistent between 9-min method and 45-min extensive separation gradient for every analyte, demonstrating the high selectivity in human pool serum.


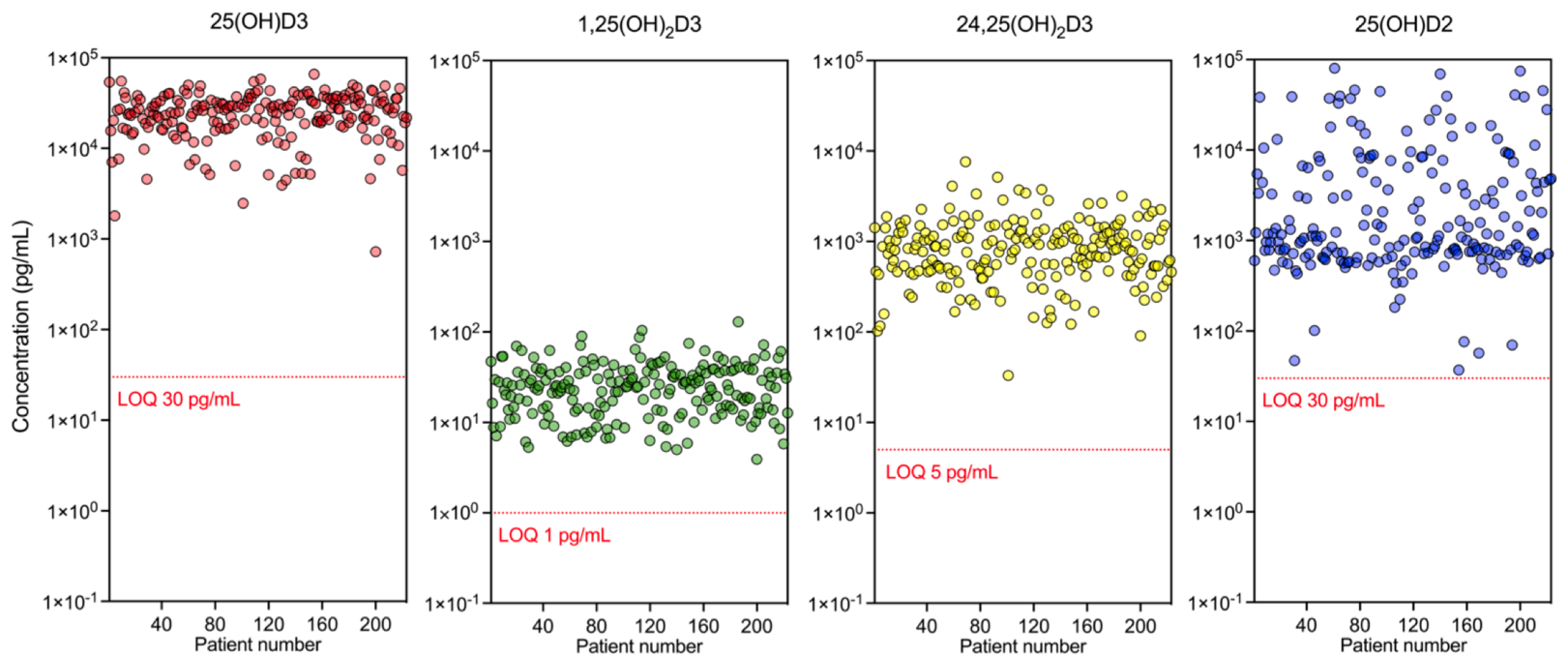


**Supplementary Figure 6** Distribution of the measured VitD metabolite concentrations in 218 multiple sclerosis patients.

**Supplementary Figure 7** Major routes of VitD metabolism to 25(OH)D, 1,25(OH)_2_D and 24,25(OH)_2_D.

**Supplementary Figure 8** The scheme for 4-phenyl-1,2,4-triazoline-3,5-dione (PTAD) derivatization reaction of measured VitD metabolites.
